# Cadmium Uptake by Wheat (*Triticum aestivum* L.): An Overview

**DOI:** 10.3390/plants9040500

**Published:** 2020-04-14

**Authors:** Tayebeh Abedi, Amin Mojiri

**Affiliations:** 1 Umea Plant Science Centre, Department of Forest Genetics and Plant Physiology, Swedish University of Agricultural Sciences, 90183 Umea, Sweden; 2 Department of Civil and Environmental Engineering, Graduate School of Advanced Science and Engineering, Hiroshima University, 1-4-1 Kagamiyama, Higashihiroshima 739-8527 Japan; amin.mojiri@gmail.com

**Keywords:** biochar, cadmium, silicon, uptake, wheat

## Abstract

Cadmium is a toxic heavy metal that may be detected in soils and plants. Wheat, as a food consumed by 60% of the world’s population, may uptake a high quantity of Cd through its roots and translocate Cd to the shoots and grains thus posing risks to human health. Therefore, we tried to explore the journey of Cd in wheat via a review of several papers. Cadmium may reach the root cells by some transporters (such as zinc-regulated transporter/iron-regulated transporter-like protein, low-affinity calcium transporters, and natural resistance-associated macrophages), and some cation channels or Cd chelates via yellow stripe 1-like proteins. In addition, some of the effective factors regarding Cd uptake into wheat, such as pH, organic matter, cation exchange capacity (CEC), Fe and Mn oxide content, and soil texture (clay content), were investigated in this paper. Increasing Fe and Mn oxide content and clay minerals may decrease the Cd uptake by plants, whereas reducing pH and CEC may increase it. In addition, the feasibility of methods to diminish Cd accumulation in wheat was studied. Amongst agronomic approaches for decreasing the uptake of Cd by wheat, using organic amendments is most effective. Using biochar might reduce the Cd accumulation in wheat grains by up to 97.8%.

## 1. Introduction

Heavy metal contamination is not only a threat to living organisms but also a global environmental concern [1]. Cadmium is generally released from industrial activities, such as refining, mining, and plastic manufacturing [2]. In addition, Cd is persistent and remains in the environment for decades [3]. Several farmlands have been polluted by metals via industrial emissions, fertilizers, and urban waste [1]. The main roles of heavy metals in plant metabolism are the basis of their involvement in reduction/oxidation procedures [4]. Cadmium toxicity is also associated with diminished plant growth, enzyme activity, and metabolism [5].

Rehman et al. [3] stated that Cd might be accumulated by plant tissue and inhibits plant growth. Cereal crop cultivars around the world can store high Cd concentrations in grains. More than 40% of Cd may be absorbed and transported to the upper parts of the plant and thus may directly (grains) or indirectly (animals) affect human health [6]. Consequently, reliable methods are needed to decrease heavy metal accumulation in crops and protect living organisms [1].

Wheat (*Triticum aestivum* L.) is the third most vital cereal in the world after rice and maize [7]. Therefore, wheat was selected to discuss cadmium uptake by plants in the current work. Almost 60% of the wheat produced globally is consumed as food [8], and wheat demand is globally expected to rise by an estimated 70% in the next few decades (2020–2050) as the human population increases and rising income levels increase household consumption [9]. In developing countries, various farmers use wastewater for irrigating crops, such as wheat, due to the fact of water shortages, thus Cd accumulation is increased in wheat grains [3]. Many studies have indicated that high Cd content in wheat is accumulated through the roots, and Cd may translocate to the shoot and grains [10]. Chunhabundit [11] reported that wheat and its products are some of the vital food contributors to dietary Cd intake by people. Different ranges of cadmium concentrations in wheat and soils have been reported by previous studies around the world (Table 1). Consequently, diminishing Cd in wheat grains is vital to alleviate its human health risks. Aside from health concerns, the effects of abiotic and biotic parameters on wheat should be also understood to ensure improved growth and produce sufficient yield. In this work, the effects of some factors on Cd uptake by wheat and the different methods of reducing Cd uptake by plants are reviewed. For preparing this review paper, several papers were studied using a selection of databases such as ScienceDirect, Springer Nature, MDPI, and Google Scholar.

## 2. Cadmium Transport in Wheat

### 2.1. Cd Entry into the Roots

Acidification of the soil increases the Cd (bio)availability for plants, and its solubility is increased by root exudates. Cadmium is present in the soil solution mostly as Cd^2+^ but also as Cd chelates [20]. Cadmium interrupts normal plant metabolism and leads to poor growth in host plants [21]. Cadmium can be transported through the roots, stems, and leaves via apoplastic and symplastic pathways [20]. Symplastic pathways are more complex than apoplastic pathways because of the role of the former in the transmembrane transport of ions [22].

Cadmium can also reach the root cells as Cd^2+^ via three main transporters: (1) zinc/iron-regulated transporter-like proteins (*ZIP*), such as *AtIRT1* and *TcZNT1/TcZIP4* transporters; (2) natural resistance-associated macrophage proteins (*NRAMP*), such as *OsNRAMP1*, *OsNRAMP5*, and *AtNRAMP6*; and (3) low-affinity calcium transporters, such as *TaLCT1* [15]. Cadmium may also enter the roots via cation channels including depolarization-activated calcium channels (DACCs), voltage-insensitive cation channels (VICCs), and hyperpolarization-activated calcium channels (HACCs). Cadmium may reach the root cells as Cd chelates over yellow stripe 1-like (*YSL*) proteins [20].

#### 2.1.1. Transporters Involved in Cd Entry into the Roots

The *AtIRT1*, a plasma membrane transporter that intercedes in heavy metal accumulation, has wide specificity for divalent heavy metals [23]. Nishida et al. [24] found that when *AtIRT1* is located in the outer layer of the root, it absorbs metals from the soil. Plaza et al. [25] stated that when *TcZNT1/TcZIP4* is placed in roots, *TcZNT1* can mediate high-affinity Zn transport along with a low-affinity Cd uptake [25].

Natural resistance-associated macrophage proteins (*NRAMP*) comprises a vastly conserved integral membrane protein family involved in iron transport in many organisms including plants [26]. The *OsNRAMP1* and *OsNRAMP5* iron transporters have been described as Cd influx transporters in the plasma membrane [27]. *AtNRAMP6* is an intracellular transporter of metals such as Cd [28].

Some divalent cations (Zn^2+^, Fe^2+^, and Ca^2+^) and Cd^2+^ are chemically similar, and Cd^2+^ is translocated by transporters for cations such as Ca^2+^ and Zn^2+^ [29]. Wheat *TaLCT1* mediates the translocation of many cations such as Ca, Cd, and K. The Cd transport movement of *TaLCT1* is inhibited by high Ca or Mg levels. *TaLCT1* possesses cation translocations with wide substrate specificity and is not specific to Cd transport [30]. Uraguchi et al. [30] stated that *TaLCT1* is specified as an influx transporter in wheat.

#### 2.1.2. Cation Channels Involved in Cd Entry into the Roots and Cd Chelate Entry into the Roots via YSL

The DACCs, VICCs, and HACCs are moderately nonselective among cations [20]. Although the regulatory mechanisms of such Ca^2+^ channels are not yet totally understood, several regulators, such as phosphorylation, reactive oxygen species (ROS), actin, and cytosolic Ca^2+^, affect their activity [31,32]. The two families of genes that code for HACCs, VICCs, and DACCs in plants may be the cyclic nucleotide-gated channel genes and the glutamate-like receptors [32]. The DACCs are the third kind of Ca^2+^ channels present in root protoplast plasma membranes. The DACCs indicate maximal activation at −80 mV (external 30 mm CaCl_2_) and display a characteristic negative slope conductance; however, they are unstable and appear infrequently [33]. The HACCs are mainly involved in guard cell closure and enable Ca^2+^ influx in response to abscisic acid, blue light, and some elicitors [32]. The HACCs may coexist with VICCs and DACCs. Thus, cells may allow Ca^2+^ influx across a wide voltage range [34].

The *YSL*, an oligopeptide transporter, transports nicotianamine (NA)–metal chelates across plant cell membranes [35]. The YSL-mediated metal transport is a mechanism possibly conserved across the plant kingdom. The *YSL* transporter is expressed only in selected tissues and induced under Fe-deficient situations. The *YSL* transporters may have key roles in metal translocations in plant tissues [36].

### 2.2. Cadmium entry into Shoots and Grains

Cadmium has high assimilability and mobility; thus, its entry into plants is possible through the roots, and it is translocated to shoots in an ionic form in the xylem and phloem over transporters and transpiration [2]. Cadmium can enter the xylem via symplastic transport and possibly through apoplastic transport under high exposure [37]. Then, Cd is loaded into the tracheids or vessel elements of the stele and translocated to the shoot [38]. Apoplastic pathways are responsible for solute transfer through the extracellular fluid and gas spaces between and within cell walls. In symplastic pathways, solutes and water are intracellularly transported and move from cell to cell over tubular channels called plasmodesmata [22].

Heavy metal ATPases (*HMAs*) may transport heavy metals across membranes and have a vital role in translocating Zn/Cd from plant roots to shoots [39]. Such integral membrane proteins apply the energy provided by ATP hydrolysis to transport metals across membranes [40]. One of the vital ATPases is P1B-ATPases (*HMA2*). The P1B-ATPases are a subfamily of the P-type ATPase superfamily, a group of ubiquitous membrane proteins that use ATP to pump cations across membranes against their electrochemical gradient [41]. Argüello et al. [42] noted that P1B-ATPases drive cytoplasmic metal efflux and contribute to maintaining cytoplasmic metal levels. They can transport Zn^2+^, Co^2+^, Cu^2+^, and Cd^2+^ [43]. Hart et al. [44] noted that Cd translocation to wheat grains might be related to phloem-mediated Cd transport to the grain. The phloem is the key Cd transport into grains. In the phloem sap, Cd may bind to the unknown 13 kDa protein and SH-compounds [27]. Uraguchi and Fujiwara [27] noted that xylem-to-phloem Cd transfer at nodes is suggested, and phloem Cd transport over a panicle neck displays genotypic variation. Such findings suggest the existence and involvement of transporters at nodes for phloem Cd transport into grains. All involved genes and transporters in the Cd uptake by wheat are listed in Table 2.

## 3. Cd Phytotoxicity and Detoxification Mechanism in Wheat

Cadmium may affect the performance of plants at several levels of biological organization, from the subcellular up to the ecosystem level [46]. At the cellular level, a range of ROS is increased when plants are subjected to Cd stress. The ROS are toxic unless eliminated quickly [47]. Cadmium toxicity results in electrolyte leakage (EL) and overproduction of hydrogen peroxide (H_2_O_2_) and malondialdehyde content in plants [10]. At the cellular level, over-accumulated Cd affects enzyme activity and changes protein structure [48]. Cadmium binding to enzymes and sulfhydryl groups of structural proteins causes the misfolding and reticence of activity or interference with redox-enzymatic regulation [49]. Several enzymes (heme, biotin, or coenzyme A) require cofactors to work suitably for metal ions and organic molecules. The displacement of one metal ion by another inhibits enzyme activities. In addition to such factors, the dislocation of Ca^2+^ by Cd^2+^ in calmodulin, a vital protein in cellular signaling, inhibits the calmodulin-dependent phosphodiesterase activity in radish [50]. At the physiological level, a set of symptoms, such as underdevelopment, chlorosis, and programmed cell death, is induced in plants when Cd accumulates at extreme levels [48]. Excess Cd negatively affects the plants’ uptake of important nutrients, such as Zn, Mn, and Fe, because of competition at the root surface. The physical symptoms of Cd toxicity are necrosis, leaf chlorosis, and plant height reduction [51]. Several symptoms of Cd toxicity are leaf roll and chlorosis, stomatal closure, and water uptake imbalance. Chlorosis may be caused by the changes in the Fe:Zn ratio due to the presence of Cd and the negative effects on chlorophyll metabolism [49].

Plants have induced some mechanisms, employing enzymes, to maintain balanced Cd levels and prevent the detrimental effects of extremely high Cd concentrations [52]. The activity of ROS-scavenging enzymes, such as superoxide dismutase, catalase, and ascorbate peroxidase, is the most vital protective mechanism to minimize metal-induced oxidative damage in many plants [53]. The nonenzymatic mechanisms of ROS detoxification may be activated; key nonenzymatic antioxidants comprise ascorbate, glutathione, flavonoids, vitamins, carotenoids, and alkaloids [52]. Aprile et al. [54] suggested that wheat with low Cd and Pb in leaves has a high expression of the gene *YSL2* which has a possible regulatory role in Cd compartmentalization in roots. The *YSL2* is localized to vacuole membranes and can transport metal–NA complexes [55]. In addition, DiDonato Jr. et al. [56] stated that *YSL2* is located in many cell types in the roots and shoots, suggesting that diverse cell types gain metals as metal–NA complexes.

## 4. Effects of Different Parameters on Reducing Cd Uptake by Plants

Different factors can affect Cd levels in wheat and can be divided into two main groups, namely, intrinsic factors (such as wheat genotypes) and extrinsic factors (such as soil properties and application of fertilizers). Stolt et al. [57] investigated the performance of different wheat genotypes in the uptake of cadmium. Their results indicated that the minimum cadmium uptake was recorded in Thasos compared with Tjalve, Topdur, and Grandur. Genetic variations in Cd accumulation occur because of differences in physiological and morphological characters of the genotypes [57].

Lu et al. [12] studied the Cd accumulation in grains by different wheat genotypes in Cd-contaminated soils. They stated that Bainong207, Aikang58, Huaimai23, and Yannong21 are good candidates of low-Cd genotypes. Low-cadmium genotypes have a large biomass and high accumulation of Cd in straw but low-Cd accumulation in grains.

Soil factors, such as pH, organic matter (OM) content, cation exchange capacity (CEC), soil texture (clay), Fe and Mn oxide content [58], and the amount of applied fertilizers, affect the Cd uptake by plants.

Soil pH is highlighted as a vital parameter that affects the quantity of exchangeable Cd in soil for plant uptake. Acidic soils contain a large amount of plant-exchangeable Cd [59]. At low pH, Cd is replaced from its sites on soil particles by aluminum ions and hydrogen and dissolved in the soil solution [60]. In acidic environments, Cd is in free Cd^2+^ ion form; however, Cd exists in other forms, such as CdHCO_3_, CdCl, hydrated CdCO_3_, and CdCl_n_^2-n^, at neutral or alkaline pH [61].

The management of soil OM helps in preventing the dynamics of soil Cd. Increasing OM frequently affects soil Cd through the adsorption of metal leasing to a decrease in plant-available Cd, whereas the weathering of organics in soil has an opposite effect [59]. Ghaley et al. [62] stated that soil organic carbon supports multiple soil features and amends the physical, chemical, and biological parameters of soil. Consequently, amending soil quality may diminish metal uptake by plants. Abedi and Mojiri [63] stated that OC in soil might affect the mobility and bioavailability of metals over redox reactions, metal–OM complexation, and competitive adsorption. 

Hucker [64] noted that CEC may play a vital role in the availability of Cd for plants. When CEC is high, a high amount of Cd is fixed in soil colloids, whereas the amount of Cd in soil solution is high at low CEC.

Nylund [60] stated that increasing clay content reduces Cd uptake by plants. Clay particles have negatively charged surfaces that might adsorb Cd. The quantity of soluble Cd in the soil solution rises with increasing clay content because of many binding sites. In addition, Liu et al. [58] stated that Fe and Mn oxides in soil may limit Cd transfer from soil to wheat.

Applying nitrogen and phosphorous fertilizers frequently has a positive correlation with Cd uptake by plants. N is a major component of various structural, metabolic, and genetic compounds in plant cells [65]. The effects of N on the uptake of Cd by plants depend on the type of fertilizer, plants, soil pH, and texture. Huang et al. [23] noted that the extreme use of nitrogen fertilizers in some areas leads to increased soil acidification, whereas acidic soil increases the Cd absorption of plants. Nitrification occurs after N is added to soil; the soil pH decreases, thereby leading to the increase in heavy metal solubility in soil [66]. Hassan et al. [65] stated that the type of fertilizer is important. Ammonium fertilizers can increase Cd concentrations in crops in comparison with NO_3_^−^ fertilizers because of pH reduction during nitrification or plant uptake of NH_4_^+^. Mitchell et al. [67] reported that wheat Cd concentrations increase with the increase in usage rate of N to 0.8 mg/Kg. However, the reported effects of N fertilizer on the Cd concentration in plants remain unclear [68]. As a component of certain molecules, nitrogen may play a role in the defense reactions of plants against many stresses [66]. Li et al. [69] reported that N application (120 kg ha^−1^) reduces wheat grain Cd concentration (0.058 mg kg^−1^ DM) and uptake (151 mg ha^−1^). Hassan et al. [65] applied (NH_4_)_2_SO_4_ to monitor the performance of plants under Cd stress. The highest chlorophyll content and photosynthetic rates and the lowest Cd content in plant were recorded in (NH_4_)_2_SO_4_-fed plants. Landberg and Greger [70] reported that the Cd concentration in wheat grain is reduced with the increase in N rate and N concentration. The decrease in Cd concentration is attributed to the dilution effect caused by the increase in biomass production.

Jiao et al. [38] found that the use of P considerably increases grain Cd concentration. Using P fertilizers may affect soil Cd availability and Cd accumulation in crops indirectly through the addition of Cd as a pollutant in P fertilizers and by influencing soil properties, plant nutrition, and growth. An increase in P results in a decrease in Zn uptake which might increase Cd uptake [22].

## 5. Agronomic Techniques for Decreasing the Uptake and Accumulation of Cd by Plants

Wheat is a major food crop cultivated in numerous areas globally with more than 650 million t grown per year. Different strategies, such as the use of bacteria, organic (compost and biochar) and inorganic (phosphate, iron oxide, gypsum, and sulfur) amendments, and other agricultural practices, have been used to reduce Cd uptake [71]. Some reported agronomic techniques in reducing Cd uptake by wheat are listed in Table 3.

### 5.1. Sulfur-Based Fertilizers

Sulfur (S) is an essential macronutrient with a crucial role in regulating plant responses to numerous biotic or abiotic stresses [72]. Shi et al. [73] reported that the application of sodium sulfate could increase dry weights of root, straw, and grain by 19%, 25%, and 25%, respectively. In addition, the application of sodium sulfate reduces grain Cd concentrations by almost 23%. However, Shi et al. [73] noted that wheat is usually cultivated in aerobic soils, and increasing sulfate concentrations in aerobic soils may increase Cd formation because CdSO_4_ is soluble in soil solutions. Furthermore, S may have contrasting effects in crops. Low S content improves crop growth, whereas high S content limits nitrogen uptake, thereby diminishing crop production; hence, a proper level of S is vital [74]. Shi et al. [73] found that the use of sodium sulfate enhances root Cd concentrations by up to 39%. Lu et al. [74] indicated that enhancing Cd uptake via root vacuoles and reducing translocation in shoots under S-treated plants may be attributed to the increasing Cd binding on cell walls, chelation, and vacuolar sequestration with the support of nonprotein thiols, phytochelatins, and heavy metal ATPases in roots; it may also prevent the expression of transporters that support Cd translocation from roots to shoots.

### 5.2. Using Si

The Si is a useful and probably important component for plants and has an essential role in plant growth and development [75]. The Si may control nutrient relationships in plants grown under environmental hazards [76]. Additionally, considerable evidence suggests that the utilization of Si in soils may alleviate Zn or Cd toxicity in various plant species [75]. Ismael et al. [61] stated that applying Se to plants under Cd stress may reduce the ethylene level and increase proline accumulation; this method may also increase the activity of glutathione peroxidase and glutathione reductase which initially relieves Cd-induced oxidative stress. The Si can enhance seedling biomass and reduce metal concentration in the shoots and roots of plant seedlings and the xylem sap flow [77]. Dong et al. [2] reported that Si utilization remarkably decreases available/oxidizable Cd in soil at 50 and 100 mg/Kg Cd levels. Naeem et al. [78] reported that Si application might reduce the Cd contents in wheat by 28%. The Si could alleviate the toxic effects of toxic metal elements on plants via external and internal mechanisms. External mechanisms comprise affecting the pH and decreasing the availability of toxic metal elements in soil, whereas internal mechanisms comprise affecting plant uptake, binding toxic metal elements in the cell walls, limiting the transport of toxic metal elements in plants, regulating protein and gene expression, and improving the defense system [2].

### 5.3. Using Zinc

Zinc may have an important role in different metabolic processes, such as photosynthesis, respiration, and assimilation of other main nutrients, and in the activation of antioxidant enzymes [79]. Zinc is an essential micronutrient that may antagonize Cd uptake by plants because of the analogous properties of both elements. The Zn-chelated amino acids are environmentally friendly and are used to enhance crop yield and quality by fortifying Zn and decreasing Cd stress [51]. Zhao et al. [80] reported that Zn might prevent Cd translocation from roots to shoots. Zinc and Cd are chemically similar and may compete for transport mechanisms during uptake and translocation in the crop [14]. Zhou et al. [81] noted that Zn has antagonistic and synergistic effects on Cd uptake by plants. Saifullah et al. [79] reported that applied Zn can decrease up to 74% of Cd concentration in the roots, straw, and grain. Applying 50 μM Zn decreases approximately 17% of Cd concentration in the shoots of wheat [81]. Rizwan et al. [51] found that adding zinc–lysine can reduce Cd concentration in plants and improve wheat growth.

### 5.4. Using Organic Amendment

Rehman et al. [3] found that organic amendments are frequently used to reduce the bioavailable fraction of heavy metals in soil. Composts and biochar are the most applicable organic amendments [82]. Organic amendments have considerable immobilizing effects on metals, because they contain humic acids that may bind with metals and decrease metal uptake by plants [83].

Compost application is a sustainable way to augment the physical, chemical, and biological properties of soil along with crop yield. Composting increases soil OM, microbial activity, plant growth, and vegetable production [82]. Moreover, 69.6% to 75% and 10.3% to 18.4% of Cd in stems and seeds of wheat, respectively, are reduced by adding 27–54 t ha^−1^ of compost [84]. Khedr et al. [85] reported that adding vermicompost can reduce extractable/available Cd by up to 98% in soil under wheat cultivation. Cadmium concentration is 34%–38% lower in spinach grown under soil amended with animal waste compost [86].

Biochar can be used to immobilize toxic traces of elements in soil due the fact of its unique properties such as high porosity, surface area, functional groups, and cation exchange capacity [87]. The reduction in Cd concentration in plants resulting from adding biochar is ascribed to the immobilization of bioavailable metals and dilution effects because of increased plant biomass [88]. Sun et al. [89] studied the effects of biochar on Cd uptake in vegetables. The results revealed that the biomass of vegetables increases remarkably by 16.9% to 519.9%, but the Cd concentrations in eggplant fruits and green pepper are reduced by 15.1% to 15.4% and 6.8% to 11.5%, respectively. When 1.5% to 5% of biochar is added to the soil, Cd concentration in wheat grains decreases by 26% to 57%, respectively [10]. Abbas et al. [71] suggested that biochar enhances soil characteristics, the Zn concentration in the shoots and roots of wheat, and the Si concentration in the soil solution, shoots, and roots. Such strategies decrease Cd accumulation in wheat grains.

### 5.5. Using Bacteria

Other agronomic techniques, such as bacteria use, have been reported in previous studies. Wang et al. [90] reported decreasing Cd (12%–32%) uptake by wheat and diminishing available Cd (15%–28%) content in rhizosphere soils using metal(loid)-resistant bacteria *Ralstonia eutropha* Q2-8 and *Exiguobacterium aurantiacum* Q3-11. Metal(loid)-resistant bacteria have been reported to enhance plant growth and diminish metal(loid) availability in soils and accumulation in plants [91]. Wang et al. [92] noted that metal-tolerant bacteria can interact directly with heavy metals to diminish their toxicity or modulate their bioavailability. Wang et al. [93] found that metal(loid)-resistant bacteria have a vital role in increasing plant biomass and metal(loid) resistance by producing siderophores, 1-aminocyclopropane-1-carboxylate deaminase, and indole-3-acetic acid. Ahemad [94] and Lin et al. [95] noted that *Rhizobium*, *Bradyrhizobium*, *Pseudomonas*, and *Stenotrophomonas acidaminiphila* can be used in reducing metal accumulation in plants. In addition, Jan et al. [96] applied *Exiguobacterium indicum* SA22 and *Enterobacter ludwigii* SAK5 to reduce Cd accumulation in rice.

## 6. Conclusions

Heavy metal contamination in soils represents a threat to the sustainability of human health due to the metals’ toxicity. Uptake and accumulation of Cd by plants, such as wheat, may pose a risk to human life. Wheat is a vital food crop cultivated in numerous areas globally. Cadmium toxicity diminishes wheat growth, mineral nutrients, photosynthesis, and grain yield. In this study, several articles were reviewed to explore the journey of Cd in wheat. The key conclusions of the present study were as follows:

1. Cadmium enters the root of wheat via transporters (*NRAMP*, *ZIP*, and low-affinity calcium transporters), cation channels (DACCs, HACCs, and VICCs), and Cd chelates via *YSL*;
2. Cadmium may easily reach plants via root uptake and translocation to shoots and grains because of its high mobility;
3. Several agronomic techniques can be used to reduce Cd uptake by wheat, the most effective of which is the use of biochar, compared to other techniques, such as using bacteria or silicon.

## Figures and Tables

**Table 1:** Cd concentration in wheat and soil globally.

Cd (mg/Kg) in Wheat; Average or Range	Cd (mg/Kg) in Soil; Average or Range	Soil Characteristics	Remarks	Area	Reference
0.14 (grain)	0.38	pH = 5.9 CEC (cmol/Kg) = 21.3 OM (%) = NR ** Clay (%) = 15.8	Yangmai16 *	The north of Zhejiang Province, China	[12]
0.12 (grain)	0.36	pH = 4.9 CEC (cmol/Kg) = 34.6 OM (%) = NR Clay (%) = 117.5	Yangmai16	The east of Zhejiang Province, China	[12]
3.17 (root) 1.11 (stem) 0.25 (grain)	2.06	pH = 7.5 CEC (cmol/Kg) = 7.6 OM (%) = NR Clay (%) = NR	Zhengmai7698	Henan Province, China	[7]
0.006 to 0.17 (grain)	0.09 to 1.0	pH = 6.6 CEC (cmol/Kg) = 18.2 OM (%) = 3.0 Clay (%) = NR	NR	Kunshan, China	[13]
0.247 (grain)	0.10	pH = 7.5 CEC (cmol/Kg) = NR OM (%) = NR Clay (%) = NR	-	Brandon, Manitoba, Canada	[14]
0.01 to 0.08 (grain)	0.21	pH = 5.3 CEC (cmol/Kg) = 31 OM = NR Clay (%) = NR	-	São Gotardo (MG), Brazil	[15]
0.95 (root) 0.60 (stem)	0.27	pH = 7.8 CEC (cmol/Kg) = NR OM (%) = 0.7 Clay (%) = NR	-	Khuzestan Province, Iran	[16]
0.01 to 0.02 (grain) 0.01 to 0.03 (grain)	3.2	pH = 7.6 CEC (cmol/Kg) = NR OM = 0.14 Clay (%) = 46	Rushan Falat	Qom, Iran	[17]
0.93 (grain) 0.16 (stem) 0.67 (root)	NR	pH = NR CEC (cmol/Kg) = NR OM = NR Clay (%) = NR	-	Lahore, Pakistan	[18]
0.003 to 0.03 (grain)	NR	pH = NR CEC (cmol/Kg) = NR OM = NR Clay (%) = NR	-	Sydney, Australia	[19]

* Local names; ** not reported.

**Table 2:** Gene families and channels involved in the Cd uptake, transport, and metabolism in wheat.

Name	Remarks	Reference
*AtIRT1*	A plasma membrane transporter. Involved in entrance of Cd into root.	[24]
*TcZNT1*	Involved in entrance of Cd to root.	[25]
*OsNRAMP1*	Cd-influx transporter in the plasma membrane. Involved in entrance of Cd into root.	[27]
*OsNRAMP5*	Cd-influx transporter in the plasma membrane. Involved in entrance of Cd into root.	[27]
*AtNRAMP6*	An intracellular metal transporter. Involved in entrance of Cd into root.	[28]
*TaLCT1*	An influx transporter in wheat. Involved in entrance of Cd into root.	[30]
*YSL*	A kind of oligopeptide transporter. Involved in entrance of Cd into root over Cd-chelates across plant cell membranes.	[35]
*P_1B_-ATPases*	A group of ubiquitous membranes. Transporting Cd from root to shoot.	[39]
*CNGC gene family*	Ca^2+^ channels in root protoplast plasma membrane. Indirectly involved in entrance of Cd into root. Responsible for coding of HACCs, VICCs, and DACCs *.	[44,45]
DACCs	Ca^2+^ channels. Involved in entrance of Cd into root.	[32]
HACCs	Ca^2+^ channels. Involved in entrance of Cd into root.	[32]
VICCs	Ca^2+^ channels. Involved in entrance of Cd into root.	[34]

* depolarization-activated calcium channels (DACCs), hyperpolarization-activated calcium channels (HACCs) and voltage-insensitive cation channels (VICCs).

**Table 3:** Reported methods for decreasing the uptake of Cd by wheat plants.

Decreasing of Cd Accumulation in Root/Stem or Straw/Grains	Cd Concentration in Wheat after Treating (mg/Kg)	Method	Remarks	Reference
48.3% (in straw) 97.8% (in grain)	0.80 (in shoot) 0.01 (in grain)	Using rice husk biochar	Mixing silicon-rich biochar with soil	[97]
54% (in root) 50% (in shoot) 65% (in grains)	2.0 (in root) 1.1 (in shoot) 0.2 (in grain)	Using co-composted farm manure and biochar	Mixing organic amendments with soil	[82]
69% (in root) 67% (in shoot) 62.5% (in grains)	12 (in root) 2.7 (in shoot) 0.15 (in grain)	Using rice husk biochar	Mixing biochar with soil	[87]
55% (in root) 51% (in shoot)	1.2 (in root) 0.7 (in shoot)	Using biochar	Mixing biochar with soil under stress conditions	[71]
57% (in grains)	0.2 (in grain)	Using biochar	Mixing biochar (5%) with soil	[10]
97% (in straw)	>0.2 (in straw)	Using limestone + biochar	Mixing limestone + biochar with soil	[98]
77% (in grains)	1.1–0.2 (in grain)	Using zinc oxide nanoparticles	Foliar application	[99]
55% to 69% (in root)	1–0.6 (in root)	Using zinc	Using ZnSO_4_ in nutrient solution	[81]
7%–24% (in root) 13%–37% (in stem) 13%–50% (in grains)	4–3 (in root) 3.8–2.2 (in stem) 0.2–0.9 (in grain)	Using zinc	Foliar application	[100]
10%–31% (in root) 27%–52% (in shoot) 33%–70% (in grains)	2.7–2.0 (in root) 1.6–0.9 (in shoot) 0.5–0.2 (in grain)	Using zinc–lysine	Foliar application	[51]
19%–64% (in root) 11%–53% (in shoot) 20%–82% (in grains)	12–5 (in root) 6–2 (in shoot) 1.1–0.3 (in grains)	Using silicon nanoparticles	Foliar application	[101]
30% (in shoot)	1.2 (in shoot)	Using inorganic silicon fertilizer	Mixing the fertilizer with soil	[102]
24% (in grains)	0.35 (in grain)	Using sodium sulfate	Mixing with soil	[73]
40% (in root)	NR	Using bacteria	Using *Ralstonia eutropha* Q2-8	[90]

* NR = Not reported.
